# Use of plasma chromogranin a epitopes vasostatin and catestatin as biomarkers for catecholamine-producing tumours in dogs

**DOI:** 10.1186/s12917-026-05996-6

**Published:** 2026-10-16

**Authors:** Josefine Prabert, Jeanette Hanson, Kumiko Ishigaki, Kazushi Asano, Katja Höglund, Mats Stridsberg, Odd Viking Höglund

**Affiliations:** 1 Department of Clinical Sciences, Swedish University of Agricultural Sciences (SLU), Box 7054, Uppsala, 75007 Sweden; 2 Department of Veterinary Medicine, College of Bioresource Sciences, Nihon University, 1866 Kameino, Fujisawa, Kanagawa 252-0880 Japan; 3 Department of Animal Biosciences, Swedish University of Agricultural Sciences (SLU), Box 7023, Uppsala, 75007 Sweden; 4 Department of Medical Sciences, Uppsala University, Uppsala, 75185 Sweden

**Keywords:** Adrenal, Adrenalectomy, Diagnosis, Medulla, Neoplasia

## Abstract

**Background:**

Differentiation of catecholamine-producing tumours from other adrenal tumours remains difficult. Chromogranin A (CgA) is a marker for catecholamine secretion in the body. An assay for quantification of the CgA epitopes vasostatin and catestatin has been validated for use in dogs but not yet evaluated in dogs with catecholamine-producing tumours. The aims were to evaluate the assays foranalysis of plasma concentrations of the CgA epitopes vasostatin and catestatin in dogs with adrenal tumours and to compare concentrations between catecholamine-producing tumours and adrenocortical tumours in dogs that were subjected to adrenalectomy. Twelve dogs with histopathologically confirmed catecholamine-secreting tumours (eleven pheochromocytomas (PCCs), one extra-adrenal sympathetic paraganglioma (PGL)) and twelve with adrenocortical tumours were included in the study. Plasma concentrations of the CgA epitopes vasostatin and catestatin were determined byradioimmunoassay (RIA), before adrenalectomy in 23 out of 24 dogs and after adrenalectomy in six of the dogs with catecholamine-secreting tumours (five PCC, one PGL). Tumour diagnosis was made by histopathology in all cases.

**Results:**

Overall, median plasma concentration of the CgA epitopevasostatin in dogs with catecholamine-secreting tumours was 0.82 nmol/L (interquartile range (IQR) 0.52 to 1.60 nmol/L), which was significantly higher(Wilcoxon rank sum exact test, P= 0.0374) than the vasostatin concentration in adrenocortical tumours (median 0.50; IQR 0.34 to 0.59 nmol/L). A subsetof dogs (5/12) with catecholamine-secreting tumours had elevated plasma vasostatin concentrations preoperatively. After successful surgery, thepreoperatively elevated plasma vasostatin concentrations decreased numerically, however without reaching statistical significance. Catestatin concentrationdid not differ between tumour types.

**Conclusions:**

Elevated plasma concentration of the CgA epitope vasostatin showed potential as a biomarker for asubset of catecholamine-secreting tumours.

## Background

Pheochromocytomas (PCCs) are neuroendocrine tumours arising from chromaffin cells localised in the adrenal medulla and referred to as intra-adrenal sympathetic paraganglioma according to recent WHO classification [1]. Tumours of chromaffin cell origin outside the adrenal glands are classified as either sympathetic or parasympathetic paragangliomas (PGLs) [1–4]. Often, the chromaffin tumours are collectively referred to as pheochromocytoma/paraganglioma (PPGLs) [5–7]. The clinical signs associated with PPGLs are often related to excessive release of catecholamines (epinephrine, norepinephrine and dopamine) [1, 5–7] and subsequent effects on the cardiopulmonary and nervous systems. Nervousness, weakness tachycardia, and hypertension can be seen [4, 8, 9]. However, the signs are non-specific, may be intermittent, or even go unnoticed [10]. Occasionally, the patients present with severe signs, e.g., as a result of myocardial injury [4, 11–13]. In addition, clinical signs can be related to the tumour mass effect and/or bleeding [3, 4, 14].

In dogs and humans, adrenal masses are visualized incidentally during abdominal ultrasonography or computed tomography for an unrelated problem, as these techniques are becoming more available [15–19]. In a study of prevalence and clinical characteristics of incidental adrenal gland masses identified in dogs undergoing abdominal CT, adrenal gland masses were found in 9% of 270 cases [17]. In a comparison of computed tomographic and pathologic findings in 17 dogs with primary adrenal neoplasia, overlapping characteristics between tumour types were found which limited the potential for reliable distinguishing based on CT alone [20]. However, the differential diagnosis of canine adrenal tumours was feasible based on triple-phase CT findings, including morphological features, CT values, and intratumoral contrast attenuation [21]. That said, differentiating PCCs from other adrenal tumours based solely on diagnostic imaging remains a challenge.

Differentiation of PCCs from, for example an “incidentaloma”, at an early stage would reduce the risk of tumour growth and invasion of *aorta* or *vena cava*, thereby improve prognosis and in addition enable a minimally invasive surgical technique [22]. Feasibility of laparoscopic adrenalectomy for resection of canine PCC was reported in a study of ten cases of unilateral non-invasive adrenal tumours [23].

Diagnosis of PCC is based on a combination of clinical suspicion, finding an adrenal mass on diagnostic imaging, specific characteristics in diagnostic imaging findings [21], and biochemical evidence of excessive catecholamine production with increased plasma and urine concentrations of catecholamine metabolites [2, 4, 24]. Ultrasound-guided fine-needle aspiration for cytological differentiation between adrenocortical tumours and pheochromocytomas has recently been shown to be relatively safe in experienced hands. However, this remains an invasive method [25, 26]. Prior to the additional confirmation with cytology or histopathology, measurement of catecholamine production is used to detect suspected PPGLs. In dogs, measurement of the metabolite of norepinephrine, normetanephrine, is preferred over measurement of epinephrine or its metabolites [27, 28] in both plasma [28, 29] and urine [30–32]. When plasma and urine concentrations were compared, it was shown in dogs that measurement of normetanephrine in urine was superior to plasma [27–33]. Norepinephrine has a relatively short half-life in blood [34]. A feature in common for the catecholamines and metanephrines is that they are secreted as a normal stress response, making interpretation difficult if samples are taken at the veterinary clinic [33, 35]. An alternative biomarker was suggested by Brömel et al., who found that undetectable concentrations of inhibin in serum before and after ACTH stimulation was highly supportive of PCC in neutered dogs with adrenal tumours [36].

Adrenalectomy is the treatment of choice to reduce the clinical signs from abnormal secretion of catecholamines and to limit the invasive local growth and metastasis that is often associated with pheochromocytomas. Furthermore, the procedure is associated with longer survival times after diagnosis [14]. Treatment with adrenoreceptor antagonists before surgery has been recommended for dogs with PCC to avoid anaesthetic complication due to sudden release of catecholamines [14, 37, 38], adding to why a diagnostic tool would be useful to ascertain diagnosis before histopathology is available. In addition, as histological characteristics are insufficient to predict tumour behaviour in PPGLs [39, 40], it would be valuable to have a biomarker that could help to distinguish between complete or incomplete removal of PCCs at follow-up.

In summary, a biomarker representing a sustained increase in noradrenalin release would be of clinical value. Chromogranin A (CgA) is such a candidate biomarker. Histologically, CgA is used as a marker for neuroendocrine tumours [1, 41]. It is a glycoprotein that plays an important role in the formation of intracellular secretory granules of neuroendocrine tissues and is co-released extracellularly together with norepinephrine and epinephrine at sympathetic stimuli [42]. CgA has been shown relatively stable in plasma compared to the neurotransmitters with a plasma half-life of 18 min in man [43], and during handling and storage conditions [44]. In man, the biomarker CgA is considered a reliable indicator of marked increase of sympathetic tone as it correlates with norepinephrine release rate [45–47], Chromogranin A degrades into several bioactive peptides including vasostatin and catestatin [48]. CgA has been used as a general biomarker for neuroendocrine tumours in humans [49–52]. Furthermore, CgA has been shown a useful prognostic biomarker for monitoring treatment outcome in humans suffering from neuroendocrine tumours [51, 53].

A radioimmunoassay (RIA) for analysis of CgA17-38 has previously been used to measure CgA in several animal species [54]. The structure and homology of the molecule, and the specific epitopes of the molecule that can be used to analyse CgA using RIA have been described, and validated for use in dogs and cats [55].

There are several studies of CgA in dogs [44, 56–66]. One study suggested that CgA may provide a useful index for evaluation of an acute stress response [56], but less reliable as an indicator for pain [44, 58, 63]. Concerning pancreatic tumours and their metastases, one study found that CgA showed promise to help diagnose masses of unknown origin as neuroendocrine tumours [61]. This was in agreement with findings in more recent case studies, where paragangliomas were immunopositive for CgA [65, 66]. Weak associations of CgA epitope catestatin has been shown in other diseases such as myxomatous mitral valve disease and bacterial uterine infection (pyometra) [60, 64].

The aims of the study were to evaluate the assays for analysis of plasma concentrations of the CgA epitopes vasostatin and catestatin in dogs with adrenal tumours, and to compare concentrations between PPGL and adrenocortical tumours in dogs that were subjected to adrenalectomy. We hypothesised that plasma CgA epitopes vasostatin and catestatin would be increased in presence of catecholamine-producing tumours and decrease after tumour removal.

## Materials and methods

### Design and setting

Stored plasma samples from patient series were used for analysis of plasma CgA epitopes vasostatin and catestatin.

### Study population

Client-owned dogs with suspected adrenal tumours referred to the Animal Medical Center of Nihon University were eligible for inclusion in the study. An informed consent was obtained from all dog owners. All dogs underwent physical examination, blood pressure measurement by an oscillometric device, ECG examination, abdominal radiology and ultrasound, triple-phase helicalcomputed tomography (CT), and blood sampling. In dogs in which an adrenal mass was identified, adrenalectomy was performed under general anaesthesia. Tumour tissue was histologically evaluated: the morphologic diagnosis was based on cellular characteristics and the anatomical location of thetumour. Immunohistochemistry was not performed. To be included in the study, dogs had to have a histopathological diagnosis of (PCC or extra-adrenal sympathetic PGL) or adrenocortical tumour (adrenal cortical adenoma or adenocarcinoma). Forcomparison, previously published data were used as reference for healthy dogs [62].

### Blood sampling and analysis

Blood was collected into EDTA tubes with EDTA-3 K, allowed to be in room temperature for approximately 10 min after which the tubes were centrifuged at 3000 rpm for 10 min. Plasma was transferred to 1.5 ml Eppendorf tubes and stored at − 80 °C for median 29 months (total range 5 to 83 months; IQR 20 to 36 months) before analysis.

Before analysis, plasma samples were transported on dry ice from Nihon University, Japan to Uppsala, Sweden. All samples were confirmed frozen on arrival. Thereafter, samples were stored at − 80 °C until batched analysis.

Analyses of the plasma CgA epitopes vasostatin and catestatin were performed by RIAs specific for catestatin and vasostatin as previously described [54, 67]. The analyses have previously been validated for use in dogs [55]. Total assay variation was less than 5%.

The plasma concentrations of vasostatin and catestatin were compared between catecholamine-producing tumours and adrenocortical tumours in samples collected before and after surgery. In addition, the vasostatin and catestatin concentrations were compared before and after adrenalectomy in dogs with catecholamine-producing tumours. The samples included six dogs with catecholamine-producing tumours of which one dog had incomplete tumour removal; the influence of was tested by performing the analysis also after exclusion of this dog.

When blood sampling was performed on more than one occasion preoperatively, the sample taken closest to surgery was chosen. When blood sampling was performed on more than one occasion postoperatively, the sample taken closest to the one-month follow-up was chosen.

### Statistical analysis

Data analysis was performed with the free software R version 4.0.3 (2020-10-10) and 4.5.3 (2026-03-11) (www.r-project.org). Data was compared with non-parametric tests (Wilcoxon rank-sum exact test, and Wilcoxon signed-rank exact test). Age, bodyweight and sex distribution were described, and compared between catecholamine-producing tumours and adrenocortical tumours. No power analysis of sample size was performed. Data are presented as medians, total range and interquartile ranges (IQR). P values < 0.05 were regarded statistically significant. Intraindividual variation in preoperative vasostatin measurements was described by the standard deviation. The correlation between plasma concentrations of vasostatin and catestatin were tested with Spearman’s rank correlation.

## Results

### Animals

The group of dogs with catecholamine-producing tumours included two Golden Retrievers, two Miniature dachshunds, two Miniature Schnauzers, two Shetland Sheepdogs, one Chihuahua, one Cocker Spaniel, one Maltese, and one Welsh Corgi. The dog with a paraganglioma was one of the Shetland Sheepdogs. In the group with adrenocortical tumours there were two Miniature Dachshunds, two Pomeranians, two Shih Tzus, one Beagle, one Chihuahua, one Golden Retriever, one Shetland Sheepdog, one Toy Poodle, and one Welsh corgi.

#### Sex and age

The dogs with catecholamine-producing tumours included seven females (four spayed), and five males (four castrated). Of the dogs with adrenocortical tumours ten were females (seven spayed), and two were males (two castrated). Median age of the dogs with catecholamine-producing tumours was 10.5 years (total range 6.4 to 14.2 years; IQR 8.6 to 11.4 years). Median age of dogs with adrenocortical tumours was 11.0 years (total range 9.6 to 15.0 years; IQR 10.6 to 12.5 years). There was no statistical difference in age between groups (Wilcoxon rank-sum test, *P* = 0.225).

#### Body weight - test between groups

Median body weight was 8.5 kg (total range 4.0 to 27.7 kg; IQR 7.1 to 14.8 kg) in dogs with catecholamine-producing tumours, and 5.9 kg (total range 3.3 to 21.8 kg; IQR 3.5 to 8.6 kg) in dogs with adrenocortical tumours. There was no statistical difference in body weight between groups (Wilcoxon rank-sum test, *P* = 0.0689).

#### Tumour location

Of the dogs with catecholamine-producing tumours, six had a tumour in the left adrenal gland, five had a tumour in the right adrenal gland, and one dog had a paraganglioma.

Of the dogs with adrenocortical tumours, eight had a tumour in the left adrenal gland, and three had right adrenal gland neoplasia.

### Vasostatin and catestatin measurements

Preoperative vasostatin and catestatin measurements were available for all twelve dogs with catecholamine-producing tumours and in eleven out of the twelve dogs with adrenocortical tumours. For one dog with adrenocortical adenoma, only one postoperative measurement was available.

Postoperative vasostatin and catestatin measurements were available in six dogs with catecholamine-producing tumours and in eight dogs with adrenocortical neoplasia, of which one dog had no preoperative measurement.

### Preoperative comparison between catecholamine-producing tumours and adrenocortical tumours

Blood samples were collected at median 1.5 days (total range zero to 43 days; IQR one to eleven days) before surgery in twelve dogs with catecholamine-producing tumours (eleven adrenal, one paraganglioma), and at median four days (total range one to 61 days; IQR one to 29.5 days) before surgery in eleven dogs with adrenocortical tumours.

#### Vasostatin

**Fig. 1:**
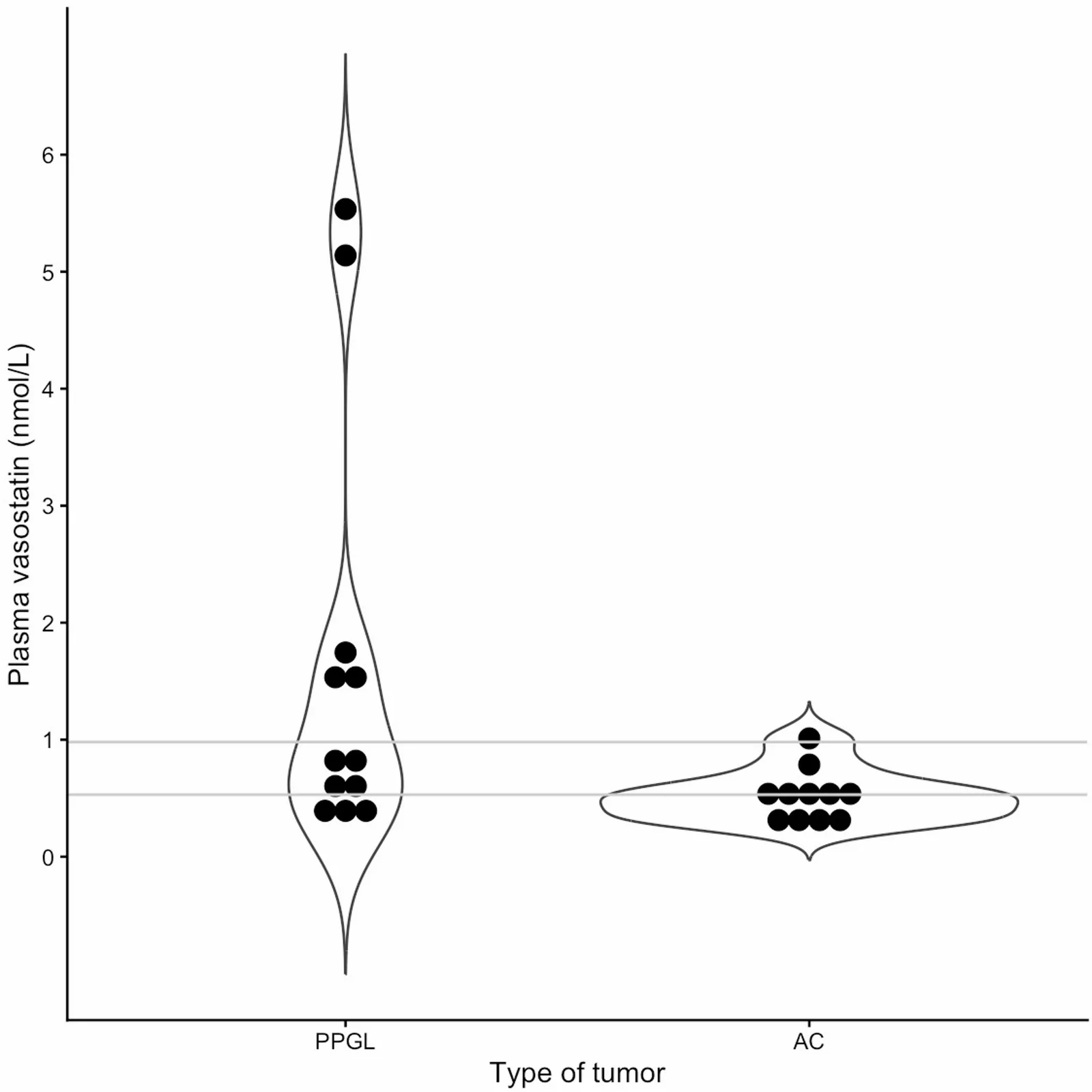
Plasma vasostatin concentrations before surgery in 11 dogs with pheochromocytomas and one dog with paraganglioma (PPGL) and 12 dogs with adrenocortical tumours (AC). Grey lines indicate the range of plasma vasostatin concentrations in healthy control dogs established in a previous study [62]

In dogs with PPGL median preoperative plasma vasostatin concentration was 0.82 nmol/L (total range 0.32 to 5.54 nmol/L; IQR 0.52 to 1.60 nmol/L). In dogs with adrenocortical tumours median preoperative plasma vasostatin concentration was 0.50 nmol/L (total range 0.28 to 1.01 nmol/L; IQR 0.34 to 0.59 nmol/L). Plasma vasostatin concentration was significantly higher in dogs with catecholamine-producing tumours than in dogs with adrenocortical tumours (Wilcoxon rank sum exact test, *P* = 0.0374) (Fig. 1).

Among the PPGL, 5 dogs (42%) had values above those of healthy control dogs (0.11 to 1.30 nmol/L) [62]. Among the adrenocortical tumours, no dog had plasma vasostatin concentrations above that of the range of healthy control dogs at the chosen time for comparison (Fig. 1).

In eight dogs, repeated preoperative measurements of vasostatin concentrations were available, 5 dogswith PPGL and 3 dogs with adrenocortical tumours. The standard deviation between intraindividual measurements of vasostatin concentrations was 0.28nmol/L with intraindividual sampling intervals ranging from 7 to 44 days.

#### Catestatin

In dogs with PPGL median preoperative plasma catestatin concentration was 0.82 nmol/L (total range 0.41 to 1.19 nmol/L; IQR 0.64 to 0.92 nmol/L). In dogs with adrenocortical tumours median preoperative plasma catestatin concentration was 0.77 nmol/L (total range 0.45 to 1.04 nmol/L; IQR 0.67 to 0.90 nmol/L). There was no statistical difference in plasma catestatin concentrations between dogs with PPGL and adrenocortical tumours (Wilcoxon rank sum exact test, *P* = 0.6505).

Of the 11 dogs with PCC, 9 dogs had plasma catestatin concentrations within the range of healthy control dogs (0.53 to 0.98 nmol/L) [62], and 2 dogs had mildly increased values (1.19, and 1.10 nmol/L respectively). Of the 12 dogs with adrenocortical tumours, 11 dogs had plasma catestatin concentrations within the range of healthy control dogs [62] and 1 dog had a mildly elevated value (1.04 nmol/L).

### Postoperative comparison between catecholamine-producing tumours and adrenocortical tumours

Blood samples were collected at median 23.5 days (total range 16 to 46 days; IQR 17.8 to 30 days) after surgery in six dogs with PPGL (five adrenal, one paraganglioma), and at median 21.5 days (total range five to 100 days; IQR 15.3 to 26.8 days) after surgery in eight dogs with adrenocortical tumours.

#### Vasostatin

Postoperative plasma vasostatin concentration did not differ between catecholamine-producing tumours (median 0.58 nmol/L (total range 0.28 to 1.7 nmol/L; IQR 0.40 to 0.66 nmol/L)), and adrenocortical tumours (median 0.54 nmol/L (total range 0.32 to 0.66 nmol/L; IQR 0.44 to 0.58 nmol/L)), (Wilcoxon rank sum exact test, *P* = 0.662).

#### Catestatin

Postoperative plasma catestatin concentration did not differ between catecholamine-producing tumours (median 0.71 nmol/L (total range 0.59 to 0.87 nmol/L; IQR 0.67 to 0.79 nmol/L)), and adrenocortical tumours (median 0.76 nmol/L (total range 0.62 to 1.00 nmol/L; IQR 0.64 to 0.86 nmol/L)), (Wilcoxon rank sum exact test, *P* = 0.662).

Of the 6 dogs with PPGL and postoperative measurements of plasma catestatin concentrations, all dogs had values within the range of healthy dogs [62]. Of the 8 dogs with adrenocortical tumours and postoperative catestatin measurements, 7 dogs had values within the range of healthy dogs [62], and 1 dog had a mildly elevated value (1.04 nmol/L).

### Comparison of samples collected before and after surgery in dogs with catecholamine-producing tumours

#### Vasostatin

Preoperative plasma vasostatin concentration in the twelve dogs with catecholamine-producing tumours was 0.82 nmol/L. In the six dogs with a postoperative sample, including one dog with incomplete removal, median postoperative plasma vasostatin concentration was 0.58 nmol/L (total range 0.28 to 1.7 nmol/L; IQR 0.40 to 0.66 nmol/L). Excluding the dog with incomplete removal, median postoperative plasma vasostatin concentration was 0.51 nmol/L (total range 0.28 to 0.67 nmol/L; IQR 0.37 to 0.65 nmol/L). Statistically, no significant difference was detected in plasma vasostatin concentration before and after surgery in dogs with catecholamine-producing tumours (Wilcoxon signed rank exact test, *P* = 0.1563).

**Fig. 2:**
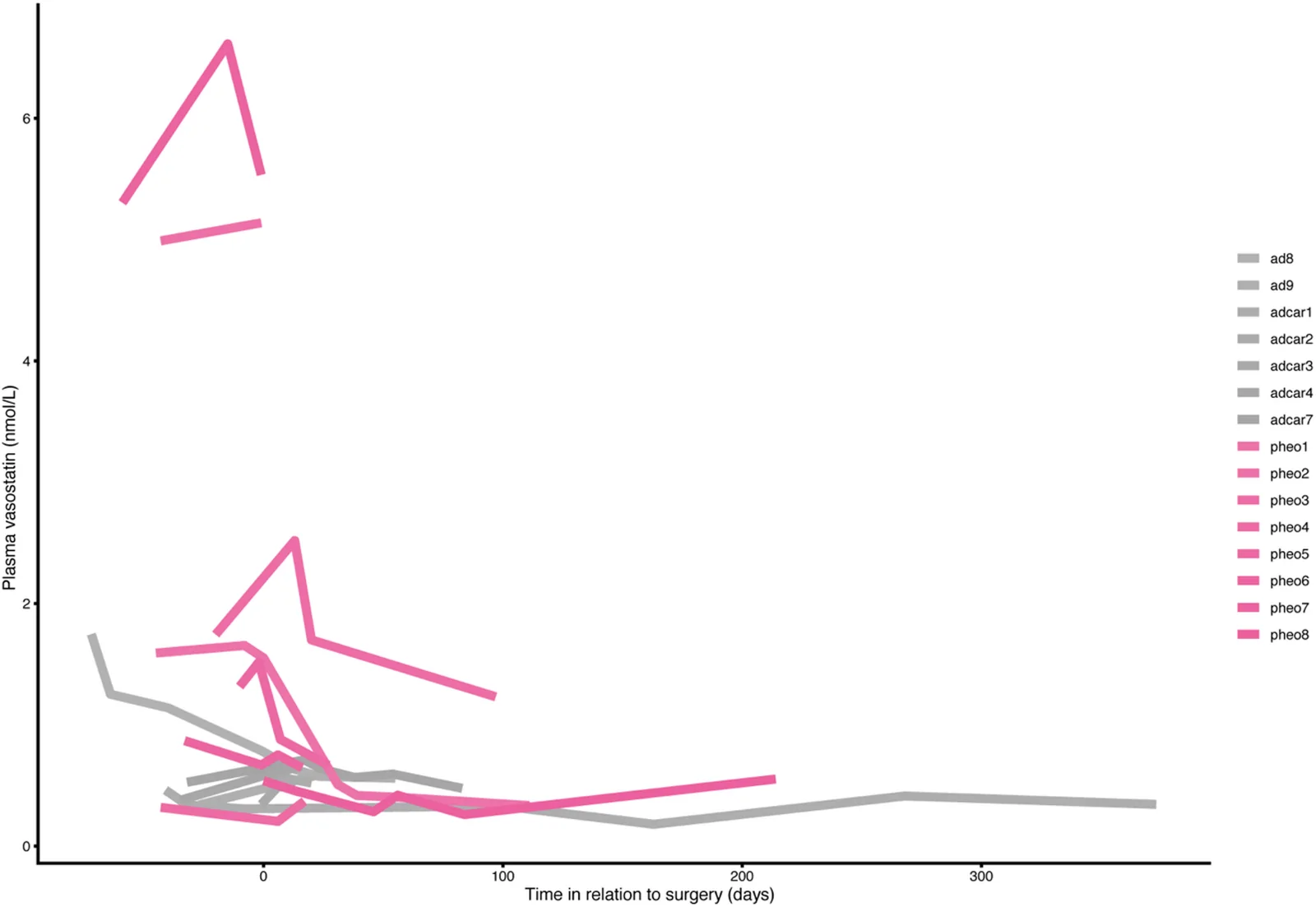
Pre- and postoperative plasma vasostatin concentrations in 7 dogs with pheochromocytomas, one dog with paraganglioma (pink), and 7 dogs with adrenocortical tumours (grey). Grey lines indicate the range of plasma vasostatin concentrations in healthy control dogs established in a previous study [62]

Of the 6 dogs with PPGL and post-operative vasostatin concentration measurements, 5 dogs had plasma vasostatin concentrations within the range of healthy control dogs (0.11 to 1.30 nmol/L) [62]. Two of these dogs had pre-operative values above the reference range. One dog had a post-operative vasostatin concentration above the range of healthy control dogs. This dog also had elevated pre-operative plasma vasostatin concentration, and an incomplete tumour removal at surgery (Fig. 2).

#### Catestatin

Preoperative plasma catestatin concentration in the twelve dogs with catecholamine-producing tumours was 0.82 nmol/L (total range 0.41 to 1.19 nmol/L; IQR 0.64 to 0.92 nmol/L). In the six dogs with a postoperative sample, including one dog with incomplete removal, median postoperative plasma catestatin concentration was 0.71 nmol/L (total range 0.59 to 0.87 nmol/L; IQR 0.67 to 0.79 nmol/L). Excluding the dog with incomplete removal, median postoperative plasma catestatin concentration was 0.70 nmol/L (total range 0.59 to 0.81 nmol/L; IQR 0.66 to 0.73 nmol/L). Statistically, no significant difference was detected in plasma catestatin concentration before and after surgery in dogs with catecholamine-producing tumours (Wilcoxon signed rank exact test, *P* = 0.4375). (Tables 1 and 2).

**Table 1:** Pre- and postoperative plasma vasostatin and catestatin concentration (nmol/L) in twelve dogs with catecholamine-producing tumours, of which 11 were PCC and 1 was PGL. Preoperative sample = sample collected closest to surgery. Postoperative sample = sample collected closest to one month after surgery

Type of Tumour	Vasostatin		Catestatin	
	Preoperative	Postoperative	Preoperative	Postoperative
PCC 1	1.55	0.51	0.41	0.81
PCC 2	5.14		1.19	
PCC 3*	1.75	1.70	0.97	0.87
PCC 4	0.32	0.37	0.86	0.70
PCC 5	1.52	0.67	0.90	0.66
PGL 6**	0.67	0.65	0.68	0.73
PCC 7	5.54		0.83	
PCC 8	0.54	0.28	1.10	0.59
PCC 9***	0.47		0.81	
PCC 10	0.40		0.52	
PCC 11	0.74		0.53	
PCC 12	0.90		0.80	

*Metastasis or incomplete removal

**Not adrenal medulla

***Recurrence of tumour postoperatively

**Table 2:** Pre- and postoperative plasma vasostatin and catestatin concentration (nmol/L) in twelve dogs with adrenocortical tumours. Preoperative sample = sample collected closest to surgery. Postoperative sample = sample collected closest to one month after surgery

Type of Tumour	Vasostatin		Catestatin	
	Preoperative	Postoperative	Preoperative	Postoperative
Adenocarcinoma 1	0.31	0.33	0.68	0.92
Adenocarcinoma 2	0.61	0.52	0.94	0.64
Adenocarcinoma 3	0.34	0.48	0.87	0.77
Adenocarcinoma 4	0.58	0.66	0.77	0.74
Adenocarcinoma 5	0.50		0.80	
Adenocarcinoma 6	1.01		0.50	
Adenocarcinoma 7	0.53	0.57	0.65	0.62
Adenoma 8	0.79	0.58	0.97	0.84
Adenoma 9	0.34	0.59	0.71	1.00
Adenoma 10	0.28		1.04	
Adenoma 11	0.47		0.45	
Adenoma 12		0.32		0.64

#### Correlation between vasostatin and catestatin measurements

The correlation between plasma vasostatin and plasma catestatin concentrations before surgery was 0.30 (95% CI -0.13 to 0.63, *P* = 0.16).

The correlation between plasma vasostatin and plasma catestatin concentrations after surgery was 0.31 (95% CI -0.27 to 0.72, *P* = 0.29).

## Discussion

The preoperative plasma concentration of the CgA epitope vasostatin was higher in dogs with PPGL than dogs with adrenocortical tumours. However, only a subset of dogs with PPGL had higher concentrations than previously established for healthy dogs. The rest of the PPGL and adrenocortical tumours had plasma vasostatin concentrations within the range of healthy dogs. After surgery, plasma vasostatin concentrations decreased to the reference range in the two dogs with preoperative elevated plasma vasostatin concentrations and successful surgery. In one dog with incomplete tumour removal, postoperative plasma vasostatin concentrations remained elevated. The two dogs with the highest preoperative plasma vasostatin concentrations, there was no follow-up, as they did not survive to discharge. Thus, although elevated plasma vasostatin concentration cannot be used to identify all PPGL it may be a useful biomarker for a subset of dogs with elevated vasostatin concentrations.

Our initial hypothesis was that the CgA metabolites vasostatin and catestatin would be elevated in dogs with PPGL because CgA is co-secreted with catecholamines, and CgA has been used as a biomarker for neuroendocrine tumours [49–52]. Indeed, plasma vasostatin concentration was significantly higher in dogs with PPGL compared to dogs with adrenocortical tumours. However, there was a considerable overlap between the groups making plasma vasostatin a poor marker to discriminate all PCC from adrenocortical tumours. Our findings are in agreement with those in humans with PPGL and other neuroendocrine tumours, in which large variations in plasma CgA concentrations have been demonstrated, from concentrations comparable with healthy controls to exceptionally high [7, 10, 43, 68]. It has been concluded that CgA is a poor marker for screening for PPGL in humans [7, 68], and the present study indicated this to hold true for plasma vasostatin in dogs.

The large variations in plasma vasostatin concentration seen in the dogs with PPGL in this study may have different explanations including normal physiological variations in catecholamine release and tumour-related characteristics. With repeated measurements in the same individual before surgery, a sampling interval ranging from 1 to 6 weeks, we demonstrated that there was little intra-individual variation in plasma vasostatin concentration between sampling occasions. Therefore, timing of sampling in relation to normal physiological variation, such as circadian variation or sampling conditions is unlikely as explanation. In human studies, such variability, although statistically significant, has only included moderate elevations in plasma CgA [43, 45]. In dogs, no circadian variation in salivary CgA was demonstrated in dogs during a 24-h period [57]. Neither did age, gender, breed, and time of day for sample collection significantly affect concentrations of plasma catestatin, vasostatin, and saliva catestatin in a previous study [62]. False positive tests in the analysis were described when CgA analysis was used in a broader population because CgA is not specific for pheochromocytomas or other neuroendocrine tumours. Chromogranin A can be secreted by tumours, endocrine and non-endocrine, and elevated concentrations of CgA can be seen in patients with liver failure, and in renal failure due to reduced elimination [48]. In humans given proton pump inhibitors, or in patients with atrophic gastritis, where there may be hyperplasia of enterochromaffin cells in the gastric mucosa which may increase CgA concentrations [52]. In general, the observed increases in CgA concentrations are usually mild to moderate although exceptions exist [52, 53] Thus, it is important to use CgA analysis in a well-defined population.

A likely explanation for the large variation in plasma vasostatin concentrations, is to be found in the variation of PPGL characteristics e.g., tumour size and biochemical activity of the chromaffin cells. The absence of increased plasma vasostatin may in some patients be explained by episodic release of catecholamine which has been described in human patients with PPGL and paroxysmal hypertension [69]. Episodic release is often related to epinephrin-secreting tumours [7]. Higher concentrations of CgA or vasostatin may correlate with the tumour mass – the larger the tumour the more secretory granules are to be released into the bloodstream upon stimulation. In humans, such correlations have been demonstrated for tumour size and metastasis in gastroenteropancreatic tumours [70]. Increased tumour size of PCC is associated with increased risk of intraoperative haemorrhage [71], increased postoperative hospital stay and postoperative complications [72]. Other possible explanations for the variability in plasma vasostatin concentration may be found in variations in metabolic activity of PPGL [73, 74]. Synthesis and storage of catecholamines and CgA vary between PPGL [75]. In histopathological examination, CgA is used as a marker for neuroendocrine tumours [40, 73]. However, CgA expression in tissue varies between pheochromocytomas [76], and poorly differentiated neuroendocrine tumours are associated with weaker CgA expression [4, 53, 77]. In addition, the difference between adrenomedullary and adrenocortical tumours may not be clear-cut, as mixed corticomedullary tumours may exist [78, 79], as do ACTH-producing pheochromocytomas [80]. A recent case report of a dog with a unilateral adrenal mass with histopathological characteristics of an adrenocortical tumour, but clinical characteristics of pheochromocytoma, including increased urinary concentrations of normetanephrines, illustrated the complexity of these cases [81].

The heterogeneity within PPGLs is further illustrated by genotypic characterization of human PPGL. Three main molecular subgroups have been described; the pseudohypoxic, the kinase signalling and the wingless type (Wnt) signalling related pathways [5, 39, 82]. It has been shown that CgA analysis has complementary diagnostic value to normetanephrine analysis in patients with pheochromocytoma and germline mutations in succinate dehydrogenase (SDH) genes SDHx-related pheochromocytomas [83], which are tumours within the so-called tricarboxylic acid cycle (TCA) aberrant cluster of the pseudohypoxic molecular subgroup of pheochromocytomas.

In dogs, genetic screening of PCC identified a variety of genetic alterations between tumours [84]. A recent metabolic profiling of PCC tumour heterogeneity was demonstrated with evidence of an altered TCA (tricarboxylic acid) cycle in a proportion of the tumours [85]. When the criteria for categorization of tumour catecholamine phenotype from human medicine was applied, 52% of the PCC were classified as adrenergic and 48% were noradrenergic [85]. In the present study, 45% of the PCCs had elevated CgA.

Although, plasma CgA has been rejected as a screening method for PPGLs in human medicine, it has been suggested to be of prognostic value in those that have elevated plasma CgA concentrations [7, 51, 75]. Very high concentrations of CgA at time of diagnosis have been associated with metastatic disease and poor prognosis [75]. In our study, there are no postoperative measurements from the two dogs with the highest plasma vasostatin concentrations. Complete tumour removal was followed by approximate 50% or more decrease in plasma vasostatin concentration to the concentrations of healthy dogs [62]. In the dog with incomplete tumour removal plasma vasostatin concentration remained elevated. Thus, plasma vasostatin may prove a useful marker for poor prognosis (if very high) and successful tumour removal in dogs with preoperatively elevated concentrations.

This study used vasostatin and catestatin as markers for CgA. However, there was only a moderate, statistically non-significant, correlation between the two which is in agreement with previous findings [62]. Vasostatin, but not catestatin, was elevated in a subset of dogs with PCCs, and therefore, vasostatin seem to be a better marker for CgA secretion than catestatin. The observed difference may be related to alterations in posttranslational processing of CgA in the PPGLs [53, 77].

The inherent bioactivity of vasostatin may also be an important aspect to take into consideration. It is known that vasostatin mediates vasodilation. Refractory hypotension is known to occur during surgery in a subset of human patients that undergo surgery for PPGL [42, 86] and has been described in dogs [87]. Hypotension is a frequent intra-and postoperative complication in patients with PPGL [72, 88]. Postoperative hypotension has been associated with higher plasma free normetanephrines and large tumour size [88]. It remains to be investigated whether the dogs with elevated plasma vasostatin concentrations are at risk of developing hypotension during surgery or not, and whether pre-treatment with an alpha-adrenergic receptor blocker such as phenoxybenzamine in these dogs may be of beneficial, neutral, or detrimental value [38].

Catestatin is also biologically active. It inhibits the release of catecholamines and has been suggested to take part in autocrine negative-feedback mechanism. In humans, it has been shown that low catestatin levels correlated with increased adrenal epinephrine secretion [42]. Further studies are needed to evaluate whether the absence of increased in plasma catestatin concentrations in the dogs that had elevated vasostatin concentrations is of pathogenetic interest.

Pheochromocytomas in humans and dogs have many similarities. An excessive catecholamine release in hormonally active PPGL causes similar clinical signs in the two species, the frequency of metastasis is similar, and they are histopathologically almost identical. Thus, the dog has been suggested as a model for study of pathogenesis of PPGL in humans [4]. The findings of the present study further support these similarities between PPGL in humans and dogs.

Our results show that plasma vasostatin is unsuitable to be used to identify all pheochromocytomas. However, a subset of dogs with PPGL had elevated plasma vasostatin concentrations. In those, plasma vasostatin may be useful as a prognostic marker. The strengths of the present study were that repeat preoperative measurements of plasma vasostatin and catestatin were available, and pre-and postoperative measurements were included. The diagnosis was based on histopathology for all the included dogs. Limitations include that the analysis was performed on stored samples. The present study focused on the differentiation of catecholamine-producing tumours from adrenocortical tumours and not on differentiating catecholamine-producing tumours from a healthy dog population. Therefore, no control dogs were included, which was a limitation; instead, previously published data were used as reference forhealthy dogs [62]. The analysis of pre- versus postoperative samples was low-powered, with only few samples included. Immunohistochemistry was not used for diagnosis, which could have increased accuracy. Furthermore, given the challenges of diagnosis, mixed cortical and medullar tumour tissue as well as additional paragangliomas or other neuroendocrine tumours may have been present concurrently but remained unnoticed. Similarly, analysis of urinary catecholamines would have increased diagnostic accuracy, which was another limitation. No Bonferroni correction was made to counteract multiple comparisons. Samples were few and sampling was not performed on a set time of day or set number of days after surgery which may have contributed to differences between dogs. However, age, gender, breed, and time of day for sample collection did not significantly affect concentrations of plasma catestatin, vasostatin, and saliva catestatin in a previous study [62]. Furthermore, the present study suggests overlapping values with what has been reported in a control population [62].

## Conclusions

The conclusion of the study is that the CgA epitope vasostatin shows promise as a preoperative diagnostic tool for a subset of catecholamine-producing tumours. More research is needed to conclude whether vasostatin could also be used as a prognostic biomarker to evaluate treatment outcome of neuroendocrine tumours postoperatively.

## Data Availability

The datasets used and/or analysed during the current study are available from the corresponding author on reasonable request.

## Abbreviations

CgA: Chromogranin A
PCC: Pheochromocytoma
PGL: Paraganglioma
PPGL: Pheochromocytomas and paragangliomas
RIA: Radioimmunoassay
